# Payments at Norwich

**Published:** 1920-09-25

**Authors:** Frank Inch

**Affiliations:** Secretary. Norfolk and Norwich Hospital, Norwich.


### Payments at Norwich.

*To the Editor of* The Hospital.

Sir,—May I draw your attention to the fact that the information appearing in your issue of September 18—namely, that patients at this hospital are charged two guineas per week—is not correct? At present the proposal that patients should pay is only under consideration by my board, and the sum suggested is not two guineas, but one guinea, per week.—Yours faithfully,

Frank Inch, Secretary.

Norfolk and Norwich Hospital, Norwich.

